# Bilateral tibial hemimelia type 1 (1a and 1b) with T9 and T10 hemivertebrae: a novel association

**DOI:** 10.1590/1516-3180.2013.1314494

**Published:** 2013-08-01

**Authors:** Victor Michael Salinas-Torres, Leticia Oralia Barajas-Barajas, Nicolas Perez-Garcia, Guillermo Perez-Garcia

**Affiliations:** I MD. Specialty Student of Medical Genetics, University Health Sciences Center, University of Guadalajara, and “Fray Antonio Alcalde” Civil Hospital of Guadalajara, Guadalajara, Jalisco, Mexico.; II PhD. Professor of Clinical Genetics, University Health Sciences Center, University of Guadalajara, and Head of Department of Special Care Clinic, Genetics Service, Integral Family Development, Jalisco, Mexico.; III MD. Professor of Radiology, University Health Sciences Center, University of Guadalajara, and Head of Department of Radiology, “Fray Antonio Alcalde” Civil Hospital of Guadalajara, Guadalajara, Jalisco, Mexico.; IV PhD. Professor of Biochemistry, University Health Sciences Center, University of Guadalajara, and Head Department of Genetics, “Fray Antonio Alcalde” Civil Hospital of Guadalajara, Guadalajara, Jalisco, Mexico.

**Keywords:** Femur, Ectromelia, Tibia, Thoracic vertebrae, X-rays, Femur, Ectromelia, Tíbia, Vértebras torácicas, Raios X

## Abstract

**CONTEXT:**

Congenital absence of the tibia is a rare anomaly with an incidence of one per 1,000,000 live births. It is mostly sporadic and can be identified as an isolated disorder or as part of malformation syndromes.

**CASE REPORT:**

A male child, born to unaffected and non-consanguineous parents, presented with shortening of the legs and adduction of both feet. Physical examination at six months of age showed head circumference of 44.5 cm (75^th^ percentile), length 60 cm (< 3^rd^ percentile), weight 7,700 g (50^th^ percentile), shortening of the left thigh and both legs with varus foot. There were no craniofacial dysmorphisms or chest, abdominal, genital or upper-extremity anomalies. Psychomotor development was normal. His workup, including renal and cranial ultrasonography, brainstem auditory evoked potential, and ophthalmological and cardiological examinations, was normal. X-rays showed bilateral absence of the tibia with intact fibulae, distally hypoplastic left femur, and normal right femur. In addition, spinal radiographs showed hemivertebrae at T9 and T10.

**CONCLUSION:**

This novel association expands the spectrum of tibial hemimelia. Moreover, this observation highlights the usefulness of this inexpensive diagnostic method (X-rays) for characterizing the great clinical and radiological variability of tibial hemimelia.

## RESUMO

**CONTEXTO:**

Ausência congênita da tíbia é uma anomalia rara, com incidência em 1 por 1.000.000 de nascidos vivos, é principalmente esporádica e pode ser identificada como um distúrbio isolado ou como parte de síndromes de malformações.

**RELATO DO CASO:**

Criança do sexo masculino, nascida de pais não afetados e não consanguíneos, apresentou-se com encurtamento das pernas e adução de ambos os pés. O exame físico realizado com seis meses de idade mostrou perímetro cefálico 44,5 cm (percentil 75), comprimento de 60 cm (percentil < 3), peso 7.700 g (percentil 50), encurtamento da coxa esquerda e as duas pernas com o pé varo bilateralhavia. Não houve dismorfismos craniofaciais, nem tórax, abdômen, genitais e anomalias das extremidades superiores. O desenvolvimento psicomotor foi normal. Os exames, incluindo ultrassonografia renal e da cabeça, potenciais auditivos evocados de tronco cerebral e exames oftalmológicos e cardiológicos, estavam normais. Raios-X revelou ausência bilateral da tíbia com fíbula intacta, hipoplasia distal do fêmur esquerdo e fêmur direito normal. Além disso, as radiografias de coluna mostraram hemivértebras em T9 e T10.

**CONCLUSÃO:**

Esta associação nova expande o espectro de hemimelia tibial. Além disso, esta observação destaca a utilidade de tal método diagnóstico barato (raios-X), caracterizando a grande variabilidade clínica e radiológica de hemimelia tibial.

## INTRODUCTION

Tibial hemimelia is a rare anomaly characterized by deficiency of the tibia with a relatively intact fibula. This defect was described by Otto in 1841 and has an incidence of one per 1,000,000 live births.^1^ Tibial hemimelia is mostly sporadic and can be identified as an isolated disorder or as part of malformation syndromes.^2^ Based on the radiographic appearance, four types of tibial hemimelia have been recognized: type 1a, with absent tibia and hypoplastic lower femoral epiphysis; type 1b, with absent tibia but normal lower femoral epiphysis; type 2, in which the tibia is distally deficient and well developed proximally; type 3, in which the tibia is proximally deficient and well ossified distally; and type 4, characterized by shortening of the distal tibia, with distal tibiofibular diastasis and normally developed proximal tibia.^3^ Patients with this longitudinal deficiency of the lower limb have unique clinical findings that vary in severity and are associated with a wide range of congenital anomalies.^4^ However, according to the International Clearinghouse for Birth Defects Surveillance and Research, congenital amelia (absence of one or both limbs) is frequently associated with intestinal defects, some renal and genital defects, oral clefts, defects of cardiac septa, anencephaly and other types of musculoskeletal defects.^5^

This report describes an infant with the novel association of bilateral tibial hemimelia type 1 (distally hypoplastic left femur corresponding to type 1a and normal right femur corresponding to type 1b) with hemivertebrae at T9 and T10.

## CASE REPORT

**Figure 1.**
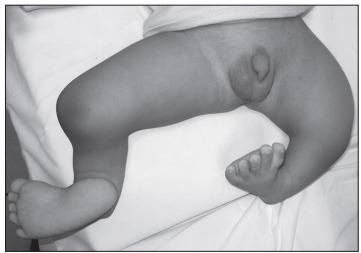
Patient showing shortening of the left thigh and both legs with bilateral varus foot.

**Figure 2.**
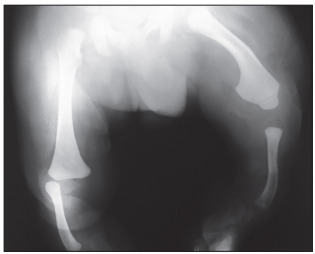
X-ray showing bilateral absence of the tibia with intact fibulae and distally hypoplastic left femur plus normal right femur.

**Figure 3.**
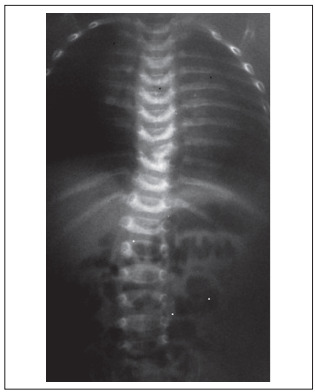
Spinal radiographs showing T9 and T10 hemivertebrae.

A male infant was referred due to shortened legs and adduction of both feet (Figure 1). Renal and cranial ultrasonography, brainstem auditory evoked potentials, and ophthalmological and cardiological examinations were normal. X-rays (Figure 2) showed bilateral absence of the tibia with intact fibulae, distally hypoplastic left femur and normal right femur. In addition, spine radiographs showed hemivertebrae at T9 and T10 (Figure 3). The karyotype with G bands (> 550 bands) was reported as 46,XY. He was the first child of healthy and non-consanguineous parents who said that he had not been exposed to mutagens or teratogens and that there was no history of affected relatives. The pregnancy had been monitored from the 10^th^ week onwards and had not presented any complications. The patient was born in the 38^th^ week by vaginal delivery with Apgar scores of 9 and 9. The birth weight was 2,800 g (25^th^ percentile) and the length was 42 cm (< 3^rd^ percentile). Physical examination at six months of age showed head circumference of 44.5 cm (75^th^ percentile), length 60 cm (< 3^rd^ percentile), weight 7,700 g (50^th^ percentile), shortening of the left thigh and both legs with bilateral varus foot. There were no craniofacial dysmorphisms or chest, abdominal, genital or upper-extremity anomalies. His psychomotor development was normal. Treatment consisting of disarticulation of the knee joint and use of a prosthesis will be attempted.

## DISCUSSION

The patient’s bilateral absence of the tibia with intact fibulae and distally hypoplastic left femur plus normal right femur prompted the diagnosis of bilateral tibial hemimelia types 1a and 1b (Figure 1 and 2); in addition, there were hemivertebrae at T9 and T10 (Figure 3). Over the past forty years, several studies have described over one hundred cases of congenital deficiency of the tibia.^1^^,^^3^^,^^4^^,^^6^ Among these patients, five had bilateral tibial hemimelia types 1a and 1b;^1^^,^^3^^,^^4^ however, none of them presented the combination of bilateral tibial hemimelia types 1a and 1b with hemivertebrae at T9 and T10. In our review of the literature, using the Medline (http://www.ncbi.nlm.nih.gov/pubmed/), Scirus (http://www.scirus.com/srsapp/), Embase (http://www.embase.com), Cochrane Library (http://www.thecochranelibrary.com/view/0/index.html), SciELO (http://www.scielo.org) and Lilacs (http://lilacs.bvsalud.org/en/) databases, we did not find any articles describing this association (Table 1). A few tibial hemimelia cases have been recorded with hemivertebrae in the lower spine.^4^

**Table 1.** Review of medical databases using the descriptors corresponding to the main features presented by the patient, conducted on August 16, 2012

Database	Search strategy	Results
Medline (http://www.ncbi.nlm.nih.gov/pubMed/)	((“Absence of Tibia” [Supplementary Concept]) OR (Bilateral Tibial Hemimelia)) AND (Hemivertebrae)	0 articles
Scirus (http://www.scirus.com/srsapp/)	((“Absence of Tibia” [Supplementary Concept]) OR (Bilateral Tibial Hemimelia)) AND (Hemivertebrae)	1 article
Embase (http://www.embase.com)	((“Absence of Tibia” [Supplementary Concept]) OR (Bilateral Tibial Hemimelia)) AND (Hemivertebrae)	0 articles
Cochrane Library (http://www.thecochranelibrary.com/view/0/index.html)	((“Absence of Tibia” [Supplementary Concept]) OR (Bilateral Tibial Hemimelia)) AND (Hemivertebrae)	0 articles
SciELO (http://www.scielo.org)	((“Absence of Tibia” [Supplementary Concept]) OR (Bilateral Tibial Hemimelia)) AND (Hemivertebrae)	0 articles
Lilacs (http://lilacs.bvsalud.org/en/)	((“Absence of Tibia” [Supplementary Concept]) OR (Bilateral Tibial Hemimelia)) AND (Hemivertebrae)	0 articles

In the present case, the finding of mid-spine hemivertebrae (T9 and T10) could be a coincidence of two independent defects. However, if this was just a random occurrence, the probability would be one in a billion (tibial hemimelia frequency^1^ = 1/1,000,000 x hemivertebrae frequency^7^ = 1/1000). Hence, this small predictive ratio supports the notion that there is a true association between tibial hemimelia and hemivertebrae, no matter what the level is.

Tibial hemimelia encompasses a heterogeneous group of disorders that are classified according to radiological and clinical signs.^3^^,^^4^^,^^6^ It may occur as an isolated anomaly or may be associated with a variety of skeletal and extraskeletal malformations such as polysyndactyly, club hand, radioulnar synostosis, bifid femur, cleft lip/palate and imperforate anus. Tibial hemimelia may also constitute a part of a malformation complex or syndrome such as the Gollop-Wolfgang complex and tibial agenesis-ectrodactyly, triphalangeal thumb-polysyndactyly, tibial hemimelia/split-hand/split-foot and Langer-Giedion syndromes.^2^^,^^8^^,^^9^ In our case, the previous workup with full ultrasonography and X-ray body scan ruled out malformations that had previously been associated with this disorder.

Although tibial hemimelia is usually sporadic, several affected families have shown either autosomal dominant inheritance with great variability and reduced penetrance or an autosomal recessive pattern with or without consanguineous unaffected parents. The tentative gene loci for tibial hemimelia are assigned to chromosome band 7q36 and 8q24, but identification of the gene(s) responsible remains elusive.^2^^,^^8^ Richieri-Costa et al. (1987) reported on 37 patients belonging to different families who had the tibial hemimelia/split-hand/split-foot syndrome. Citing other authors, they suggested that the maximum risk to the offspring from an affected person coupled with an unaffected person is 8.6% and that the maximum risk to a sibling of an isolated patient is 12.5%.^8^^,^^9^

## CONCLUSIONS

In conclusion, X-ray imaging enables excellent assessment of tibial hemimelia and its associated skeletal malformations,^3^^,^^4^^,^^6^ in addition to its ready availability and minimal cost. The radiological features described in the present case expand the spectrum of malformations associated with tibial hemimelia and further illustrate the usefulness and sensitivity of such an inexpensive diagnostic method. Thus, physicians need to be acutely aware of the great clinical and radiological variability of tibial hemimelia. Newly available genomic technologies from biological models may begin to offer more answers regarding the causes of tibial hemimelia in the near future.
